# System Responsiveness to the Psychosocial and Mental Health Needs of Children in Ethiopian Primary Schools: The Case of Gondar City, Northwest Ethiopia Needs

**DOI:** 10.3389/fsoc.2021.573306

**Published:** 2021-03-05

**Authors:** Yemataw Wondie, Tesfaye Tadele

**Affiliations:** Department of Psychology, University of Gondar, Gondar, Ethiopia

**Keywords:** system responsiveness, teachers, policies, primary schools, psychosocial conditions, mental health condition, Ethiopia

## Abstract

**Background:** Ethiopia is the second most populous nation in Africa with children and adolescents constituting more than 40% of the population. Evidence shows the onset of significant degrees of mental illnesses is detectable in this age range. For such early identification to be made there should be a system responding to those needs.

**Objective:** The objective of this study was to explore the extent to which the education system is responsive to the psychosocial and mental health needs of children in primary schools through putting appropriate professionals in place, raising teachers’ awareness and putting in place viable policies and guidelines.

**Methods:** An exploratory qualitative study was conducted in public and private primary schools in Gondar city Data was collected through focus group discussions from seventeen participants drawn from both schools and key informant interviews with two experts from the zonal Department of Education. A thematic qualitative data analysis was employed. Themes were identified with the help of the Nvivo 12 plus software.

**Results:** We found teachers’ mental health awareness is very low with parameters such as magnitude, case identification and support. There is an exception in terms of causal attributions of mental illness that matches with scientific literature. Psychosocial support and mental health resources are not available and schools do not provide capacity building mental health trainings for teachers which might help them to identify, handle and make referrals of mental health cases. We also found the Ethiopian education policy and other guidelines do not address the issue of mental health at primary school level.

**Conclusion:** The Ethiopian education system is not responsive to the psychosocial and mental health needs of children in primary schools.

**Implications:** Arresting minor impairments before they become major disabilities is vital. Investing in childhood mental health enables a healthy and productive society to be cultivated. The Ethiopian education system should therefore respond to the psychosocial and mental health needs of children in primary schools.

## Introduction

Studies show 20% of children and adolescents have at some point experienced mental health issues in the form of social, emotional and behavioral difficulties (Loades and Mastroyannopoulou, 2010). Specifically, 50% of mental health disorders begin by the age of 14 and 75% by the age of 24 (Kessler et al., 2005). Mental illness among young people have significant association with bad school performance, a low level of social interaction, trouble with the law, higher school dropout and teenage pregnancy rates (Breslau et al., 2008).

Early identification and treating of children with mental illnesses would reduce the personal and societal burden of these disorders and offer early chances for treatment in addition to protecting the children from a possibility of worsening academic and social functioning (Miller, 2014).

The school community is an unprecedented opportunity to improve the lives of children and adolescents. The World Health Organization (WHO) urges a comprehensive mental health program be part of a comprehensive school health program including health instruction at all grade levels, easily accessible health services, a healthful nurturing and safe environment and interaction with families and community organizations (WHO, 1994). The school system and teachers are significant parties to activities aimed at promoting childhood mental health and treating mental disorders, especially in low- and middle-income countries (Patel et al., 2008).

In contrast to parents who are biased observers, teachers are neutral and can detect the behavioral changes of their students if they are trained to do so (Parikh et al., 2016). Teachers can serve in the early identification of mental illnesses, referral to specialists, reducing stigma, and enhancing the awareness of their students about mental illnesses. Many school-based mental health interventions require teacher implementation and selective or indicated interventions often involve teacher referral (Greenberg et al., 2001). However, there is often a lack of knowledge about mental health among school teachers, which impairs their ability to identify those suffering from mental disorders, as well as to educate and handle them (Walter et al., 2006). The lack of information creates insecurity and complicates the teachers’ management of everyday situations involving mental disorders (Soares et al., 2014).

In Ethiopia, there has not been yet a national representative study about the prevalence of mental illness. Estimations state that the average prevalence of mental illness in Ethiopia are 18% for adults and 15% for children (Sathiyasusuman, 2011). Such shortage of data about child mental illness is mainly due to severe shortage of child mental health professionals in the country (Desta et al., 2017). It is believed that in Ethiopia, where resources are limited, schools can have a significant role in mental health service provision given limitations of formal mental health care (Desta et al., 2017).

Recently, there have been commitments shown by the Ethiopian government such as development of a mental health strategy and the allocation of new funds for the rollout of that strategy across the country (World Health Organisation, 2016). However, promoting mental health in schools is still not addressed. The present study tried to explore the educational system’s degree of responsiveness to the psychosocial and mental health needs of children in primary schools considering schools in the public and private sectors in Gondar northwest Ethiopia.

The specific objectives of this study were 1) to examine the level of awareness of school teachers about the psychosocial and mental health conditions of school children; 2) to explore the psychosocial and mental health support resources available in primary schools, and 3) to explore the education system’s responsiveness to the psychosocial and mental health needs of school children in those schools.

## Methods

### Research Design

We employed a thematic qualitative approach by which qualitative data was collected using a couple of focus group discussions (FGDs) and with in-depth interviews, useful in generating wide and rich understanding of the participants’ experience and beliefs (Mishra, 2016).

### Participants

A total of nineteen participants were involved in this study of which seventeen were primary school teachers and two education experts working in the Central Gondar Zone Education Department. Of the primary school teachers, eight were from the University of Gondar Community School and the remaining nine were from a public school known as Abiyot Firie Primary School.

### Data Collection Tools

Semi-structured FGD’s and interview guides were used to collect data from the study participants. The FGD guide addresses the mental health awareness among school teachers, psychosocial support available in the primary schools, and the system’s respond to the psychosocial and mental health needs of school children which are all addressed in the objectives of the study. The interview guide mainly addresses system and structural issues in relation to psychosocial support and mental health in primary schools at district, zonal, regional, and national levels.

### Procedures

We followed a series of steps to prepare both the FGD and interview guides. First, the FGD and interview guides were prepared in English; second, they were translated to Amharic, the national language of Ethiopia, and then back translated to English with the help of experts in both languages. Third, the FGDs and key informant interviews were conducted and audio-taped upon securing informed consent from the participants. Finally, the authors transcribed the audio information and translated them to English. The FGD participants from both schools were selected purposively on the basis of their long time services in teaching. We had two FGDs one constituted eight participants and the other nine. Each FGD was one time and took an hour and half. Two key informant education experts were also selected purposively based on their expertize responsible for supervising primary school education. Both the key informant interviews were one time and took an hour each.

### Data Analysis

We employed thematic qualitative data analysis. Themes were identified with the help of Nvivo 12 plus software. We followed a number of steps in making the analysis. First, the raw data was entered into Nvivo 12 plus to map the patterns of the data set. Second, meaningful units of codes were extracted from the data sets that include mental health awareness, available psychosocial support and mental health resources, and system responsiveness to psychosocial and mental health needs of school children. Finally, the analysis was made based on these themes corresponding to the research questions.

### Ethical Considerations

Ethical clearance was obtained from the Department of Psychology, University of Gondar. Permission was also granted from both of the school principals and the zonal Education Department head. Informed consent was obtained from all the participants and both of the key informants.

## Findings

### Characteristics of the Study Participants

A total of 19 individuals took part in this study, eight from the University of Gondar (UoG) Community School, and nine from a public school in Gondar called Abiyot Firie Primary School. Apart from this, two experts from the Central Gondar Zone Department of Education also participated. Table 1 shows details of the participants’ characteristics.

**TABLE 1:** Socio-demographic characteristics of study participants.

Participants’ affiliation	Sex				Educational status
	M	F	T	Mean age (SD)	
Community school	4	4	8	33 (3.29)	Bachelor to masters degree
Public school	2	7	9	39 (9.37)	Diploma (Grade 12 + 2)
Education department	1	1	2	49 (12.73)	Bachelor degree
Total	7	12	19	37 (8.91)	

The majority (63%) of the study participants were female, their educational status ranging from diploma holder (grade 12 + 2) to masters degree level. Specifically, while the educational status of the participants from the Community School ranged from bachelor to masters degrees, those from the public school were all diploma holders.

### Thematic Analysis of the Data

Initially, we had seven categories into which the data obtained from the participants fell, namely: 1) prevalence of mental illness, 2) mental illness identification, 3) causal attribution and treatment options, 4) experiences in supporting children with mental illness, 5) availability of training opportunities on school mental health, 6) organizational structure for referrals, and 7) availability of policies and guidelines on psychosocial well-being and mental health at macro and school levels.

**FIGURE 1:**
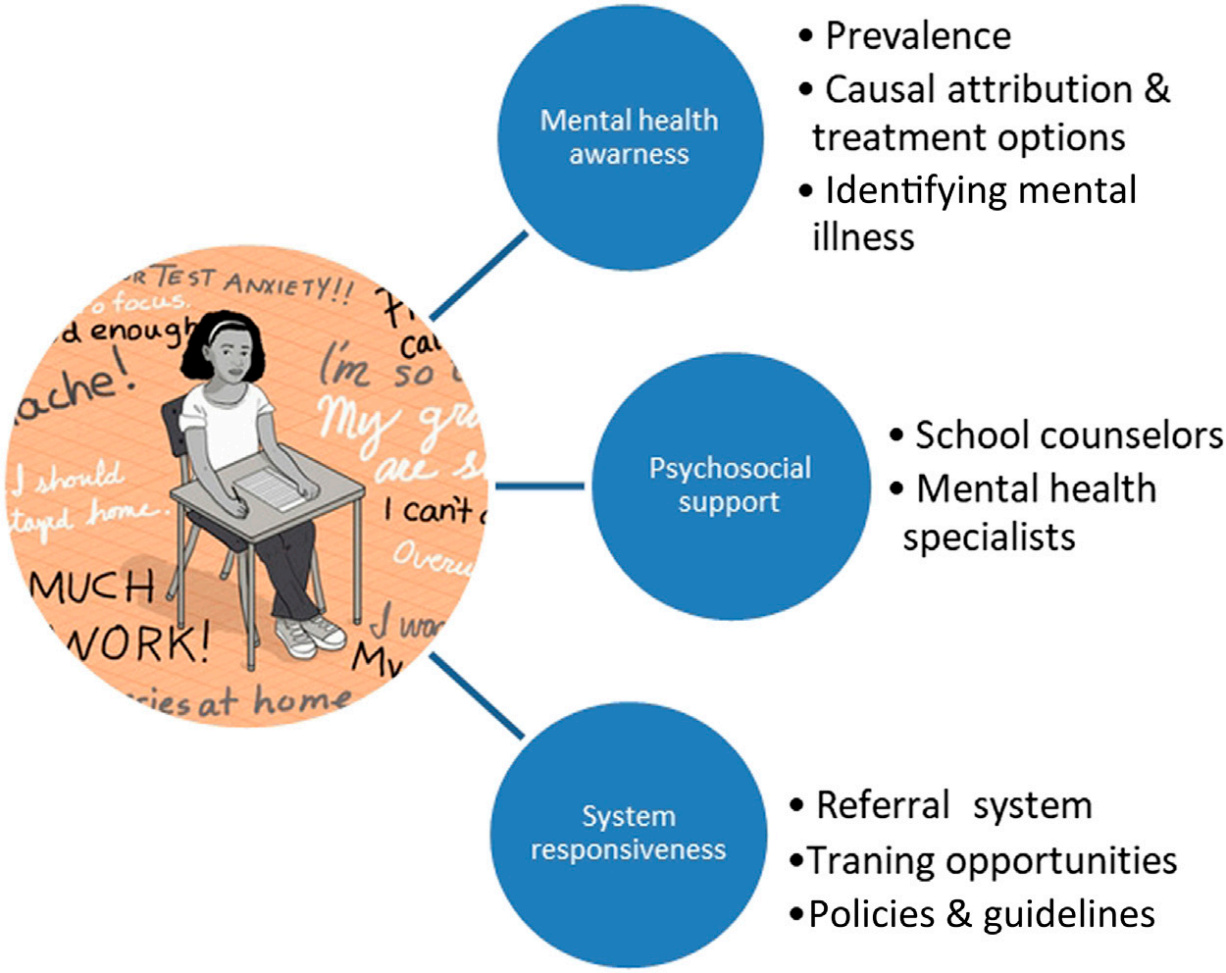
Themes explored on system responsiveness to psychosocial and mental health needs of school children.

On further inspection and categorization of the data supported by Nvio 12 plus software, we had three distinct themes in line with our research questions. Accordingly, 1) mental health awareness, 2) available psychosocial support and mental health resources, and 3) system responsiveness to psychosocial and mental health needs of school children (see Figure 1).

### Teachers’ Awareness About Psychosocial Issues and Mental Health Among School Children

The participants’ awareness about the psychosocial and mental health conditions among school children was assessed using FGDs and key informant interviews made on mental health awareness related issues. These include the extent to which the participants know about the extent of psychosocial and mental health problems among school children, their experience in identifying children with these conditions in and out of the classroom, and their knowledge about the causal attributions and treatment options they may consider for children with such conditions.

***Extent of the problem:*** Almost all the discussants underlined children with psychosocial and mental health conditions are prevalent in their respective schools. However, students with such conditions are seen to be children with special needs. What came to most participants’ minds were children with epilepsy, those with intellectual disability, children with sensory impairments and so on. For instance, one of the discussants stated the following.

“There are students with intellectual disability in every classroom. They repeat classes for five to six consecutive years. Because we are not able to help them to get to the desired level, they do not show any progress.”

We found no difference between the FGD teacher discussants from the community and public schools when it comes to such understanding in regards to psychological problems and mental illness.

***Identifying children with psychosocial and mental health conditions:*** The participant school teachers’ awareness about psychosocial mental health conditions of their students was also assessed in terms of the mechanisms they employ to identify children with such conditions. Accordingly, various strategies are employed, most of which use observation. We tried to classify and analyze such observed conditions and identified them in terms of thought-related, emotion-related, and behavior-related conditions. However, the participants were not able to give such labeling to the various psychosocial and mental health conditions of school children.

In terms of *thought-related conditions*, the discussants identified children who lack concentration and are not able to follow the teacher instruction, some strangely request to go home soon after they arrive at school because they wrongly perceive it was going home time while it was actually too early. Teacher discussants also reported some children have low achievements compared with their classmates consequently they repeat classes; some have a problems of understanding and some forget and are unable to remember things. Related with thoughts, school teachers also reported some students who cut their peers with sharp objects which appears to be “normal” for them because they don’t regret their actions. Some were also observed giggling or singing while classes were conducted.

The participants were also able to observe children having *emotion-related conditions*. These children to be observed with lower in emotions such as experiencing sadness, social isolation and loneliness. More specifically, such children prefer to stay passive and hence are not able to move on and play with other children; don’t participate in the classroom responding to questions when asked; they don’t communicate when spoken to; unable to maintain eye contact while talking (shyness); prefer to stay in their own world rather than paying attention to their surroundings; and they tend to be more passive compared with their peers. On the other hand, some children are observed to have high emotion-related conditions, showing anger and impulsiveness.

The discussants’ whether in or out of the classroom observations and reports of their respective students’ conditions could fall under behavioral problems. Some such *behavior-related conditions* include being resistant to follow rules, repeating words a teacher might have spoken to students in a class and continuing that behavior for days, kicking classmates and spitting on others, throwing backpacks through the window, repeated crying spells, splashing water on others while class is being conducted, being hyperactive, and irrationally screaming, standing up or laying on the floor during class time.

***Causal attributions of psychosocial and mental health conditions:*** The teachers’ awareness of such conditions was explored on their knowledge of the various causal factors to which school children’s conditions could be attributed. Asked about the possible causes of these conditions, the discussants brought a number of issues into the FGDs that could be seen as *natural vs. environmental causes*. While the predominant cause the discussants considered natural is believed to be genetic, those considered environmental are a wide range of conditions that could happen during pregnancy and after birth.

Pertaining to *prenatal environmental causes*, the participants believe anything that could happen to the mother or the fetus during pregnancy could cause psychosocial and mental health conditions on children sooner or later. These include lack of a balanced diet during pregnancy, taking drugs during pregnancy; a pregnancy in the mothers later years, accidents during pregnancy, and the socio-emotional condition of pregnant mothers.

Concerning postnatal causes, the discussants considered a number of issues including but not limited to corporal punishment that could be experienced at home, school or both, restricting children to stay behind closed doors (not allowing children to play with others or an inability to contact their parents), lack of love and attachments with parents, when parents knowingly or unknowingly give unbalanced attention to one of their children compared with their siblings, trauma due to accidents such as falling or getting stabbed, familial disintegration such as divorce, and lack of a balanced diet, a condition by which children may not get essential nutrients important for brain development.

Unlike the case in the general population, we did not find all the school teacher participants attributing psychosocial and mental health conditions of their students to demonic possessions. We can, therefore, fairly state that the participants’ attribution of causality sounds more scientific. Under the theme of mental health awareness, the participants unveiled the significant magnitude of the problem.

***Treatment options:*** Finally, as part of the teachers awareness on psychosocial and mental health conditions of school children, the discussants were asked about the treatment options they thought to be considered. The treatment options they considered include health professionals, psychologists, special classroom arrangements to be taught by trained professionals, spiritual interventions such as Holy Water treatment, and provision of unconditional love and affection for children with psychosocial and mental health conditions across all levels (family, community, school).

### Availability of Psychosocial Support and Mental Health Resources for School Children

Participants were asked about psychosocial supports made and what resources are available in order to make correct intervention for children with psychosocial and mental health conditions. In both schools, there is no school counselor or mental health specialist assigned to help children with their conditions. Given this, the participant school teachers stated the day-to-day challenges they are facing with a sense of intense despair. The following anecdotes portray such situations.

“There is no as such significant support we provide to children with mental illness in our school. The maximum we can do is sending them to their parents or the school nurse.”

Bothered by such challenges, one of the participant teachers also stated her situation as follows.

“There is nothing I could do to the children with mental illness in the classroom. Sometimes because they don’t go along with other students in terms of attending my class, I let them to stay out and get relaxed until I finish teaching.”

Another participant from the public school FGD group stated the alternatives they employ to address children’s conditions in the school system where there are huge number of students in every classroom.

“We try to support children with mental illness as much as possible. If we don’t have enough time because of the large number of students, we team up these children with academically good students. We also try to give them special attention and support them. If they are much less than our expectations (poor handwriting, for example), we discuss the problem with parents and try to find better ways for improvement.”

Teachers also reported urging students to bring their parents so they could advise them for a better follow up; and using school clubs such as Civic and Ethical Education Club to generate some relevant resources toward helping students cope with their conditions.

### System Responsiveness and Organizational Structure

Given all the challenges mentioned, we wondered how the school system in particular and the micro level education organizational structure could be responsive to those challenges. In this connection, we asked all the participants a series of questions for their reflections.

***Capacity building trainings:*** One is about their opinion on the importance of getting *capacity building trainings* on psychosocial and mental health conditions of school children, and whether or not they have ever taken such a training so far. On this, all the discussants in both schools equally stated the importance of school psychosocial and mental health training. However, they have never taken such training. A participant stated this as follows.

“No question about the importance of getting such training because important others for children/students next to their parents/families are teachers … who could be able to know and identify the mental health conditions students might have. Unfortunately, there is no any training given to us in our school related to mental health so far. Therefore, if training on mental health is given to teachers, we can help identifying problems among our students.”

This shows that the school system is not responding to the psychosocial and mental health needs of school children through providing capacity building trainings to the existing teachers let alone hiring the respective professionals in the field.

***Organizational structure:*** The other issue raised and discussed with the participants was whether there was any *organizational structure* such as a tripartite relationship (i.e., school administration, teachers and parents) available to respond to the psychosocial and mental health needs of school children. Discussants from both FGD arrangements stated there was no such functional organizational structure that could respond to the needs of those school children. The following anecdotes clearly portray what members of the FGDs had to say during the discussion. For instance, a discussant from the community school critiqued what was lacking from each stakeholder.

“There is no established system on the relationship among students, school administration and teachers with respect to mental health. Parents don’t want to believe and accept psychosocial and mental health problems of their children. They do not also want to give information to teachers/the school on the mental health status of their children. In addition, the main focus of the school administration is on the academic performances of the students rather than showing readiness and acknowledging what other problems they might have.”

Another discussant from the same school also underlined that the relationship among teachers, parents and the school administration around mental health issues of the students is negligible. The school doesn’t request any information regarding the health/mental health status of the students for admission, which clearly demonstrates schools do not have any intentional actions toward addressing the psychosocial and mental health conditions of their students.

On the other hand, we have realized there are some of functional clubs in the public school through which teachers and students come together to support children in need. For example, one of the participants from this school stated the following.

“We have a Civic and Ethical Education Club in our school. The club tries to identify children with mental illness/intellectual disability and we give them some sort of guidance and counseling and tutorial and follow up to the needs of these children.”

Even then, it is difficult to say that such endeavors of kind-hearted teachers and students could address the needs of children with psychosocial and mental health conditions. Another participant from the community school believes in the importance of having the right person in the right place.

“Mental health issue does not have any responsible body in our school. It is hard to say that we are fully serving our students with mental illness in the absence of a trained professional assigned to the clubs or committees. There should be a person with the necessary knowledge, skills and attitudes who could better serve them.”

***Referral system:*** What we have tried to explore so far shows that schools do not have mental health resources to support school children in need. With this in mind, we asked the discussants in both schools whether there is a structured *referral system* in place for children in need of professional counseling or mental health treatment. All the discussants said there is no established system in place, but there are individual-teacher information-based referrals in the schools, clearly the lack of cultured and shared referral system.

For instance, there were participants who stated “*Whenever we encounter such kind of students, we only inform their parents to make the necessary care and follow up*.” Another participant from the community school says “*Previously*, *we used to refer students with mental illness to the school’s guidance and counselor. We don’t have such a professional in our school currently.*” Still another discussant from the same school stated “*Whenever I have students with mental or any other illness, I refer them to the school nurse. I think the school nurse will further refer them to treatments she may consider appropriate for them*.”

***Policies and guidelines on psychosocial support and school mental health conditions:***

We tried to explore the issue of whether or not policies, guidelines and follow ups on the psychosocial and mental health conditions of school children are available through key informant interviews. As experts in the field of psychology, we have two related concerns. On one hand, most of the proliferating higher education institutions (HEI) in Ethiopia graduate young professionals at least at a bachelor degree level, these young graduates increasingly end up with no jobs. On the other hand, given the fact that the onset of most psychosocial conditions and mental illness is during childhood, especially primary school age, whether Ethiopian primary schools have started hiring mental health professionals such as psychologists which was not the case in the past, when we were in those schools.

We raised a series of related questions for our key informants. These included 1) the degree of attention given to the psychosocial and mental health conditions of children in the primary schools across all (i.e., national, state, zonal and district) levels, 2) whether there is any structural support given to this, 3) whether there is any commitment on the ground in assigning trained professionals in the field, and 4) whether they have envisioned to put the service in place that is supported by policy or guideline.

In our conversations with key informants the issue of children’s psychosocial and mental health conditions and related academic performance appears to be neglected. For instance, one of the key informants’ states:

“Among those given attention in our education policy is ensuring equity. Ensuring equity means that paying attention to those segments of the population who didn’t get attention from the government or other responsible bodies in the past. In the education sector, there are international agreements about people with special needs that we need to address in providing with access to education such as those with hearing impairment, visual impairment, delayed mental development, learning disability, and those who are not able to attend education due to lack of parental support.”

The key informants emphasized the issue of equity but not quality, to which the Ethiopian education policy is committed to address through letting children, regardless of disability, gender and socio-economic status access education. However, we found the psychosocial and mental health conditions, regardless of any disability, gender or socioeconomic status have remained unaddressed. The other key informant also underlined this.

“There is no attention given to the psychosocial and mental health conditions of children at the primary school level so far unlike the issue of special needs education. There is no organizational structure for such a service at this level either except at secondary education level. Even then, we have guidance and counseling professionals in only the 11 out of the 48 high schools in our zone let alone having such a provision at the primary schools level.”

According to the key informants, the government has the intention to consider professional psychosocial support at the primary school level. However, there is much ignorance and resistance on the relevance of such a provision and services with district level officials where the policy and structure allows for high schools. One of the key informants also considered his long term experience in the education system which in his view is stagnant in consideration of this relevant service for the whole development of children. He stated the following.

“… I was teaching in the elementary and high school levels for 38 years. I then became education expert/officer. When I generally look at the education system, there is no consideration of supporting children with mental illness in the primary school. This is emanated from ignorance and lack of attention to the importance of psychosocial well-being of children at this level of education.”

Yet the key informants believe things need to be done based on capacities and priorities. By capacity, they meant the government has not the budget or human power to allocate the required resources at the primary school level and by priority, they stated the government’s policy direction is to give equitable access to children with special needs. However, this is paradoxical from the objective of the present study which is to explore whether the education system in Ethiopia is responsive to the psychosocial and mental health conditions of children in the primary schools witnessing the abundance of graduates in the helping profession such as psychology remaining jobless; and such important conditions of children with and without special needs remaining unaddressed.

Finally, the FGD participants of both schools recommended what should be done for schools to have psychosocial support and mental health services. Schools, from admission to graduation, need to demonstrate their intention to address the psychosocial and mental health conditions of their students. There should be a functional tripartite engagement among the school administration, teachers and parents to work together toward the psychosocial well-being of school children. They also highlighted the importance of raising awareness of school mental health and developing skills supporting children with these conditions through professionally guided capacity building trainings. Apart from this “schools could do it if they are intentional,” issue, the participants believe that there should be no policy or structural barrier that preventing primary schools having psychosocial support and mental health services facilities by trained professionals.

## Discussion

As educated in the Ethiopian school system on one hand, and as professionals in the field of psychology with a fair knowledge of the current states of schools in various nations around the world on the other, we wanted to explore the extent to which the education system is responsive to the psychosocial and mental health needs of children in their primary schools in Ethiopia. In this connection, our objectives were 1) to examine the level of awareness of school teachers about the psychosocial and mental health conditions of school children; 2) to explore the psychosocial and mental health support resources available in primary schools, and 3) to explore the education system responsiveness to the psychosocial and mental health needs of school children in those schools.

Teachers’ awareness about the psychosocial and mental health conditions of school children was assessed by their relative perception of the degree of the problem, their experience in identifying children with the conditions, and their knowledge about causal attributions and treatment options they may consider for such children. All the participant school teachers perceived the magnitude of mental health problems among school children has become increasingly prevalent. This is in line with previous reports indicating mental illness as the leading non-communicable disorder accounting for 12.45% of the burden of diseases (Uznanski and Roos, 1997). Recent reports also show the average prevalence of mental illness in Ethiopia as 18% for adults and 15% for children (Sathiyasusuman, 2011). However, children with psychosocial and mental health conditions for the participant teachers in our study appear to be children with special needs such us developmental delay, learning disabilities and sensory disabilities showing that primary school teachers do not have mental health literacy. This is similar to other low income countries (Mahmoud et al., 2018).

Participants of the present study tried to identify primary school children with specific psychosocial and mental health conditions. However, they were not able to label such conditions into cognitive, emotional, and behaviorproblems.

Albeit, teachers and the school system, are regarded as important parties in identifying, treating and promoting childhood mental health problems, especially in low and middle income countries, (Patel et al., 2008). Evidence shows that early identification and treatment of children with these conditions would help reduce the personal and societal burden and provide early opportunities for treatment and protect school children from worsening academic and social problems (Miller, 2014). Teachers and other school staff can play such important roles of task-shifting (Lancet Global Mental Health Group, 2007) in the identification and intervention of mental health difficulties when they are equipped with sufficient mental health literacy (Whitley et al., 2013). If well trained, teachers are reported to be neutral observers compared with parents (who have a biased view of their children) and can detect behavioral changes of their school children (Parikh et al., 2016).

Our study participants’ causal attribution of mental illness in school children could fall into natural vs. environmental factors. By natural, the discussants meant genetic factors playing roles for mental illness inherited from parental lines. On the other hand, those considered environmental are a wide range of factors that could happen to the child during pregnancy and after birth. This is similar to a study conducted in South Ethiopia (Kerebih et al., 2016) where a range of such factors were reported. Unlike the case in the general population, however, we did not find all the school teacher participants attributing psychosocial and mental health conditions of their students to demonic possessions.

Regarding availability of psychosocial and mental health support resources, there are no school counselors or mental health specialist assigned to help children with their conditions. A guidance counseling service is regarded as a necessary component of the school system by which each student is enabled to develop a positive self-image and actualize their adjustment needs that leads them into the future (Egbo, 2015). Provision of such an important service to children in primary schools is not new in other African countries such as Zambia (Zimba and Changala, 2018), Kenya (Wambu and Fisher, 2015), and Nigeria (Egbo, 2015).

Trained professionals are not assigned to assume the role of providing guidance counseling and mental health services for children in primary schools, the education system needs to seek strategies to fill this gap. To this end, the discussants and key informants reflected upon the extent to which the school in particular and education system in general are responsive to the psychosocial and mental health needs of school children with their level possible. We considered a number of factors such as capacity building trainings, organizational structure for a functional alliance among school administration, teachers and parents, availability of referral system, and viable policies and guidelines in favor of psychosocial support and mental health in primary schools.

Regarding the capacity building training for school teachers, the result revealed that all the discussants in both the schools considered the importance of school psychosocial and mental health training as high. However, So far they have taken no such training. Lack of information on student mental health jeopardizes teachers’ responsibilities and creates insecurity while complicating the management of everyday situations involving mental health problems of school children (Soares et al., 2014). Experiences from elsewhere indicate that school-based mental health interventions requiring teacher implementation and selective or indicated interventions often involve teacher referral (Greenberg et al., 2001). However, lack of knowledge about mental health among school children impairs school teachers’ ability of case identification, intervention, and the referral of cases (Walter et al., 2006).

Considering a tripartite relationship among school administration, teachers and parents to alleviate the behavioral and mental health problems of school children, the result revealed that such a functional alliance does not exist. On the other hand, there are a couple of functional clubs in the schools such as “Charity Club” and “Civic and Ethical Education Club” through which teachers and students come together to support children in need. Scaling up and task-shifting of mental health services in low and middle income countries are recommended by WHO (Lancet Global Mental Health Group, 2007; Desta et al., 2017), and have already been practiced in various settings through mental health literacy given to teachers, parents and other school staff (Whitley et al., 2013; Daniszewski, 2013).

A lack of mental health literacy and knowledge about school mental health among teachers affects not only their ability to identify and handle children with psychosocial and mental health conditions but also their ability to make appropriate referrals (Walter et al., 2006). In the present study, the participants unveiled that there were no organized referral system nor were they aware on where to send children with mental health conditions. Perhaps, some refer such children to the school nurse or call parents to take care of their children. This is different from the range of alternative referrals teachers in south Ethiopia would like to make (Kerebih et al., 2016). The difference could be due to teachers in the present study were asked the practical referral system their respective schools use and their actual experience in making referrals compared to a “wish list” of referrals teachers would do in the south Ethiopian study.

Finally, we tried to explore viable policies and guidelines in favor of psychosocial support and mental health in primary schools at a local, regional (i.e., State) and national (i.e., federal) levels. We did this through key informant interviews with the education experts. The result shows school mental health in the Ethiopian primary schools remains unaddressed in the Ethiopian Education Policy (Ministry of Education, 1994) and the recently issued education roadmap (Teferra et al., 2018). Due emphasis is given to children with special needs to get access to education. This is happening while mental health interventions at the primary school level are considered to be so crucial given a significant degree of psychosocial and mental health conditions begin (Breslau et al., 2008) and are prevalent at this level (Kessler et al., 2005). Paradoxically, young university graduates in psychology who could play significant roles in school mental health remain jobless mainly due to the impermissible and non-responsiveness of the education system from taking in those graduates to assume such relevant responsibilities in primary schools. If conditions continue as is and won’t improve children with and without special educational needs will not have the right to get psychological and mental health services.

## Conclusion

The study tried to explore the Ethiopian system of education in regards to degree of responsiveness to the psychosocial and mental health service needs of primary school children through exploring awareness of school teachers, availability of school mental health resources and whether viable policies and guidelines exist. From this qualitative study, we conclude that mental health awareness among the participant school teachers were very low in terms of labeling various psychosocial and mental health conditions in terms of children’s experience as cognitive, emotional, and behavioral. Their experience of identifying such conditions was not supported by mental health literacy. On the other hand, we found the teachers’ knowledge of causal attribution to be more of scientific than divine-related unlike the general population. While mental health resources such as psychologists and mental health specialists are non-existent in these primary schools, we found no mental health service scaling up and task-shifting capacity building trainings for school teachers. Finally, the provision of school mental health services in primary schools are not addressed in any of the available policy documents in Ethiopia.

## Limitations

Qualitative data was collected from only a community school and a public school in Gondar, Ethiopia. This makes the findings not to be generalized to a wider cultural and geographic context. Participants were approached under the partial lockdown due to the COVID-19 global pandemic. Such a condition might have put them not at ease during the FGDs and the key informant interviews.

## Data Availability

The raw data supporting the conclusions of this article will be made available by the authors, without undue reservation.
